# Drivers of the dynamics of diazotrophs and denitrifiers in North Sea bottom waters and sediments

**DOI:** 10.3389/fmicb.2015.00738

**Published:** 2015-07-21

**Authors:** Haoxin Fan, Henk Bolhuis, Lucas J. Stal

**Affiliations:** ^1^Department of Marine Microbiology, Royal Netherlands Institute for Sea ResearchYerseke, Netherlands; ^2^Department of Aquatic Microbiology, Institute of Biodiversity and Ecosystem Dynamics, University of AmsterdamAmsterdam, Netherlands

**Keywords:** N_2_ fixation, denitrification, *nifH* gene, *nirS* gene, North Sea

## Abstract

The fixation of dinitrogen (N_2_) and denitrification are two opposite processes in the nitrogen cycle. The former transfers atmospheric dinitrogen gas into bound nitrogen in the biosphere, while the latter returns this bound nitrogen back to atmospheric dinitrogen. It is unclear whether or not these processes are intimately connected in any microbial ecosystem or that they are spatially and/or temporally separated. Here, we measured seafloor nitrogen fixation and denitrification as well as pelagic nitrogen fixation by using the stable isotope technique. Alongside, we measured the diversity, abundance, and activity of nitrogen-fixing and denitrifying microorganisms at three stations in the southern North Sea. Nitrogen fixation ranged from undetectable to 2.4 nmol N L^−1^ d^−1^ and from undetectable to 8.2 nmol N g^−1^ d^−1^ in the water column and seafloor, respectively. The highest rates were measured in August at Doggersbank, both for the water column and for the seafloor. Denitrification ranged from 1.7 to 208.8 μmol m^−2^ d^−1^ and the highest rates were measured in May at the Oyster Grounds. DNA sequence analysis showed sequences of *nifH*, a structural gene for nitrogenase, related to sequences from anaerobic sulfur/iron reducers and sulfate reducers. Sequences of the structural gene for nitrite reductase, *nirS*, were related to environmental clones from marine sediments. Quantitative polymerase chain reaction (qPCR) data revealed the highest abundance of *nifH* and *nirS* genes at the Oyster Grounds. Quantitative reverse transcription polymerase chain reaction (qRT-PCR) data revealed the highest *nifH* expression at Doggersbank and the highest *nirS* expression at the Oyster Grounds. The distribution of the diazotrophic and denitrifying communities seems to be subject to different selecting factors, leading to spatial and temporal separation of nitrogen fixation and denitrification. These selecting factors include temperature, organic matter availability, and oxygen concentration.

## Introduction

The microbial biogeochemical cycle of nitrogen transfers atmospheric dinitrogen gas (N_2_) to bound nitrogen in the biosphere and back to N_2_ (Revsbech et al., 2006). Dinitrogen fixation is the reduction of N_2_ to ammonia, which is subsequently assimilated into amino acids and proteins to synthesize biomass. There are two processes that return bound nitrogen back to atmospheric N_2_. Denitrification reduces nitrate or nitrite stepwise to dinitrogen (Zumft, 1997), while anaerobic ammonium oxidation (anammox) also produces N_2_ gas, using nitrite as oxidant (Jetten et al., 1998). Nitrification is the aerobic oxidation of ammonia to nitrite and nitrate, substrates for both denitrification and anammox. Denitrification and anammox are anaerobic processes. The former seems to be quantitatively more important than the latter in most habitats, although in certain environments anammox has been shown to out rate denitrification (Kuypers et al., 2003, 2005). It is unknown whether or not in any microbial ecosystem the nitrogen cycle is functional at the same spatial and temporal scales or that they are (partly) occurring separated. Here, we investigated denitrification and dinitrogen fixation in the North Sea in order to answer this question.

Denitrification mainly takes place in the sediments of the seafloor of the coastal shelf (Seitzinger and Giblin, 1996; Codispoti et al., 2001). Coastal shelf seas are therefore major sinks for bound nitrogen and have been estimated to account for up to 67% of the global denitrification (Codispoti et al., 2001). The North Sea is such a coastal sea located on the European continental shelf, bordered by the United Kingdom in the west and Belgium, The Netherlands, Germany, Denmark and Norway in the east. Denitrification in the North Sea bottom sediments varies from 0.9 to 255 mmol m^−2^ year^−1^ (Brion et al., 2004) and is the most important sink of nitrogen under hypoxic conditions (Middelburg et al., 1996).

Denitrification is carried out by a variety of different bacteria. The key intermediate step during denitrification is the reduction of nitrite to nitric oxide, which is catalyzed by either NirS (Cytochrome cd1) encoded by *nirS* or NirK (copper nitrite reductase) encoded by *nirK* (dissimilatory nitrite reductase). Nitrite reductase genes have been used as molecular markers for denitrification in natural environments. Phylogenetic analyses revealed the diversity of denitrifying bacteria in a variety of habitats such as soil (Prieme et al., 2002; Throbäck et al., 2007), estuarine sediments (Santoro et al., 2006), marine sediments (Braker et al., 2000; Hannig et al., 2006), and seawater (Jayakumar et al., 2004; Castro-Gonzalez et al., 2005; Oakley et al., 2007). Studies on denitrification in the North Sea were limited to rate measurements (Law and Owens, 1990; Lohse et al., 1993, 1996) or geochemical modeling (Van Raaphorst et al., 1990, 1992; Middelburg et al., 1996; Seitzinger and Giblin, 1996; Hydes et al., 1999). Not much is known about the diversity of denitrifier communities in the North Sea. In order to assess the denitrifier community composition we targeted the *nirS* gene at three sites in the North Sea that differed in depth and seafloor sediment composition.

N_2_ fixation occurs in the pelagic as well as in various benthic habitats including photosynthetic microbial mats (Severin and Stal, 2008), sea grass sediments (McGlathery et al., 1998; Herbert, 1999), and estuarine and shallow marine sediments (Fulweiler et al., 2007; Bertics et al., 2013). N_2_ fixation is an important process for nitrogen depleted freshwater and brackish water bodies and in the warmer (sub)tropical ocean where it is driven by heterocystous (freshwater and brackish) and non-heterocystous (filamentous and unicellular; tropical ocean) cyanobacteria. Surprisingly, N_2_ fixation is largely absent from temperate marine waters. N_2_ fixation seems to be negligible in the North Sea, including the seafloor, although cyanobacterial microbial mats in intertidal sediments are a notable exception (Severin and Stal, 2008). Apparently, the nitrogen demand of the North Sea waters and sediments is covered from run-off and wet- and dry deposition (Brion et al., 2004), although this does not seem to cover the demand and therefore does not fully explain the absence of diazotrophs. There have been some reports of heterotrophic N_2_ fixation in coastal waters (Gardner et al., 2006; Fulweiler et al., 2007; Bertics et al., 2010). Thus, diazotrophic microorganisms other than cyanobacteria may have been overlooked and might play a more important role than previously thought (Dang et al., 2013).

The reduction of N_2_ to ammonia is catalyzed by nitrogenase, an enzyme complex composed of dinitrogenase and dinitrogenase reductase and that is similar among all diazotrophs (Sohm et al., 2011). The gene encoding nitrogenase reductase, *nifH*, is commonly used as a marker of diazotrophs in ecological studies (Zehr and Capone, 1996). In shallow marine sediments, N_2_ fixation is mainly attributed to sulfate-reducing bacteria (Bertics et al., 2010, 2013; Brown and Jenkins, 2014). Hitherto, N_2_ fixation has not been measured in the southern North Sea. In general, little information is available on the importance of biological N_2_ fixation in temperate coastal waters (Brion et al., 2004).

The aim of this study was to measure N_2_ fixation and denitrification in the bottom sediments of three different stations in the southern North Sea during three seasons. The diazotrophic and denitrifying communities and their activities were determined at the same time together with metadata of a range of environmental variables. With this research we elucidated: (1) N_2_ fixation does occur in the southern North Sea bottom sediments and in the water column; (2) the identity of the microorganisms involved in N_2_ fixation and denitrification; (3) the spatial and temporal trends of N_2_ fixation and denitrification in the bottom.

## Materials and methods

### Study area and sampling

**Figure 1:**
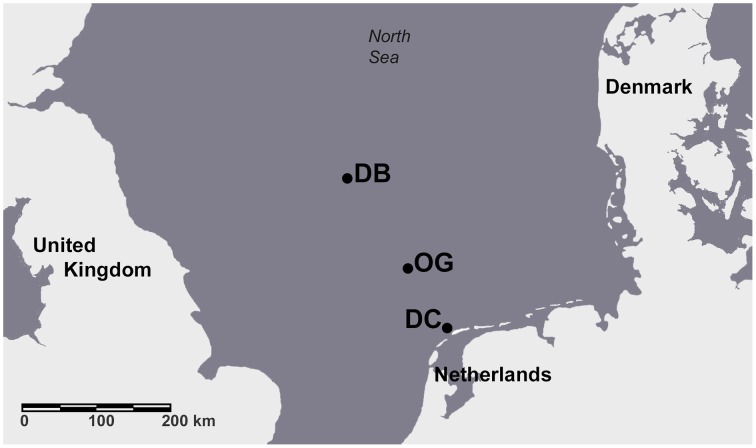
**Map of the North Sea with the three sampling stations marked**.

Four cruises were completed aboard the R/V Pelagia between November 2010 and August 2011. The study sites were located along the “Terschelling Transect” in the North Sea and the geographical coordinates are given in Bale et al. (2014) (Figure 1). The Dutch Coast station (DC) is located in the well-mixed water of the coastal area where the sediments are typically sandy and have a low organic content. The Oyster Grounds station (OG) is a large circular depression in the central southern North Sea. The bottom sediments at OG are muddy sands and the organic carbon content is an order of magnitude higher compared to the DC and DB (follows) sites. The water column at the Dogger Bank station (DB) is 30 m deep. The bottom sediment at DB is sandy and contains a low amount of organic matter. The properties of the sediments at the three sampling stations are described in Bale et al. (2014). Sampling of the bottom sediment and the overlying water was performed using a box corer. Intact sediment cores were collected from the box corer using custom-made cores (20 cm long × 5 cm i.d.) and were used to measure denitrification and N_2_ fixation. Physicochemical parameters were measured in the water column and in the sediment as described by Bale et al. (2014).

### Denitrification and N_2_ fixation

The rates of denitrification and N_2_ fixation were measured by the ^15^N stable isotope technique. The procedures for measurement and calculation of the rate of denitrification were described previously (Bale et al., 2014). The dissolution of ^15^N_2_ in medium for the measurement of N_2_ fixation was performed according to Mohr et al. (2010) with some modifications. Briefly, 500 ml ASW (artificial seawater) (NaCl 20.5 g, Na_2_SO_4_ 3.4 g, KCl 0.58 g, KBr 0.084 g and H_3_BO_3_ 0.022 g, MgCl_2_.6H_2_O 10.2 g, CaCl_2_.2H_2_O 1.1 g in 1000 ml Milli-Q water) was degassed by vacuuming for 45 min (KNF Neuberger, type N726.3 FT.18) in an ultrasonic bath. The degassed ASW was transferred to 300 ml Schott bottles until overflow and sealed after which 3 ml ^15^N_2_ (98%) were injected. The bottle was shaken overnight before it was used for enriching samples with ^15^N_2_. Fifty milliliter of the ^15^N_2_-enriched ASW was added to 450 ml ASW, which was subsequently used for the slurry incubations. For the measurement of N_2_ fixation, sediment from the top 5 cm was homogenized to slurry (equal volumes of sediment and ASW). Ten milliliters of slurry were put into 50-ml serum bottles, which were subsequently filled with the ^15^N_2_-enriched ASW. The bottles were sealed with butyl stoppers while avoiding air bubbles. The bottles were incubated for 24 h in the dark at *in situ* temperature. Incubations were terminated by removing the overlaying water and freeze the sediment at −20°C. *In situ* denitrification rates were measured as described in Bale et al. (2014). Nucleic acids were extracted from the top 5 cm of the sediment collected by box cores from the three stations. The sediment samples were taken from the box cores using 15-ml plastic screw cap tube and stored immediately at −80°C until analysis.

### Nucleic acid extraction, PCR, cloning, and sequencing

DNA and RNA from the water column were extracted according to the procedures described by Bale et al. (2013). DNA and RNA were extracted from the sediments using the MoBio UltraCLEAN soil DNA and RNA kit (MoBio Laboratories, Inc., Carlsbad, CA, USA), according to the manufacturer's instructions.

The quantity and quality of RNA were determined and checked by Nanodrop spectrophotometer (Nanodrop ND1000, Thermo Scientifica, Wilmington, DE, USA) and agarose gel electrophoresis, respectively. The RNA extracts were immediately treated with RNase free DNase I (Deoxyribonuclease I, Amplification Grade, Invitrogen Corporation, Carlsbad, CA, USA). DNA contamination was checked by PCR using the DNase-treated RNA extract as template. After the DNase treatment and the confirmation of the absence of DNA the RNA concentration and quality were checked again. The DNA-free RNA was reverse transcribed to cDNA using Superscript II Reverse Transcriptase and random primers (Invitrogen Corporation, Carlsbad, CA, USA) following the manufacturer's manual. Controls were run that either lacked reverse transcriptase or the RNA extract and should not give a product. The synthesized cDNA was kept at −20°C until further use.

For amplification of *nifH* and its transcripts we used a nested PCR with inner primer pair nifH 1 (5′ TGY GAY CCN AAR GCN GA 3′) and nifH 2 (5′ ADN GCC ATC ATY TCN C 3′) (Zehr and McReynolds, 1989) and outer primers nifH 3 (5′ ATR TTR TTN GCN GCR TA 3′) nifH 4 (5′ TTY TAY GGN AAR GGN GG) (Zani et al., 2000). The PCR conditions have been described in Severin et al. (2010).

Both *nirS* and *nirK* were initially tested, however, we subsequently only targeted *nirS* as this gene is preferentially found in marine sediment, while *nirK* is more common in soil (Braker et al., 2000). Fragments of *nirS* were amplified using the primer pairs cd3aF and R3cd (Throbäck et al., 2004). PCR conditions for this primer pair were 2 min at 95°C, 35 cycles of 50 s 95°C, 50 s 53°C, and 50 s at 72°C, followed by a final extension of 10 min at 72°C. PCR products were checked on a 1% agarose gel. PCR products were cloned using the TOPO-TA cloning kit with the pCR2.1 vector and TOP10 competent cells (Invitrogen, Carlsbad, CA, USA) following the manufacturer's instructions. Transformants (99 for *nifH* and 50 for *nirS*) were randomly picked from each clone library and screened by PCR using T3 and T7 vector primers following the recommended PCR conditions (Invitrogen, Carlsbad, CA, USA). PCR products were purified and checked as described by Severin et al. (2010) and sequenced with the T7 vector primer using ABI PRISM 3130 Genetic Analyzer (Applied Biosystems, Foster City, CA, USA).

### Quantitative real-time PCR

Quantitative real-time PCR (qPCR) analyses were run on a Corbett Rotor-Gene 6000TM (Corbett Life Science, Sydney, Australia). The copy numbers of *nifH* and *nirS* were determined by primers nifH_q1 (5′CgYggYgTTATCACYgCYATCAACTT 3′) and nifH_q2 (5′ CgAAACCRCCRCARACAACgTC 3′) (Tm = 53°C) and by primer pair cd3aF and R3cd (Throbäck et al., 2004) (Tm = 53°C), respectively. For the quantification of the *nifH* gene, primers nifH_q1 and nifH_q2 were designed by aligning 200 sequences (main groups as revealed by phylogenetic analysis) obtained from cloning the PCR products amplified by using the Zehr and Zani primers. In order to confirm the specificity, 48 amplification products from sediment samples were cloned and sequenced. All of these amplicons encode a *nifH* gene and the majority was identical and clustered amongst those listed in Table S1. DNA (dilution 1:10) and cDNA samples were run in triplicate. Standard curves were made by dilution series of linearized plasmids (quantified by Nanodrop before using as standard for quantification) containing the target genes and were run parallel to each analysis. Non-template controls were also included in each run. The reaction mixture (15 μl) contained 7.5 μl of Absolute™ QPCR SYBR® Mix (Thermo Fisher Scientific, Rockford, IL, USA), 0.2 pmol/μl primers, 1 μl template and sterilized MQ water. Cycling conditions were as follows: 95°C 15 min, 45 cycles of 15 s 95°C, 20 s Tm, and 20 s at 72°C, followed by melting curve analysis (50–95°C). The standard curves spanned a range from 22 to 2.2 × 10^6^ copies per μl for the *nifH* and 12 to 1.2 × 10^6^ copies per μl for the *nirS*. PCR efficiencies (E) and correlation coefficients for *nifH* were 85% and *R*^2^ = 0.99 and for *nirS* were 81% and *R*^2^ = 0.99.

### Sequence and statistical analysis

Sequences were manually checked, aligned, and translated using MEGA 6 (Tamura et al., 2013). Neighbor-joining trees were produced and the reliability of the phylogenetic reconstructions was evaluated by bootstrapping (1000 replicates). The Prodist program within Phylip v.3.6 (Felsenstein, 2005) generated the distance matrix files of amino acid sequences. These files were used to calculate the non-parametric richness and diversity estimators and to determine the differences in nucleic acid sequences applying the program Mothur (Schloss et al., 2009). Operational Taxonomic Units (OTUs) were defined as a 5% difference in amino acid sequences for the purpose of community analysis. The principal coordinate analyses were generated using Mothur. The coverage of the clone library was calculated as C = [1−(n/N)] × 100, where n denotes the number of unique OTUs (95% amino acid cutoff was used) and N denotes the total number of sequences examined (Good, 1953). The Pearson correlation test was performed using software the SigmaPlot™ v12.0.

### Nucleotide sequence accession numbers

Sequences were submitted to NCBI (accession numbers KP959349–KP959733).

## Results

### Physicochemical parameters

Physicochemical parameters in the water column were described in Bale et al. (2013). Ammonium concentrations in the pore water of the bottom sediment (5 cm) ranged between 4.1 and 23.4 μM and were highest in August at all stations (Table 1). Nitrate concentrations were between 4.9 and 46.2 μM and were always highest in February. Pore water nitrite concentration ranged between 0.2 and 1.3 μM and was highest in August at DC. The concentration of phosphate in the pore water ranged between 1.0 and 3.0 μM and the highest value was detected in August at DB.

**Table 1:** **Sediment pore water nutrients and bottom water temperatures (top 5 cm)**.

	**February**			**May**			**August**		
	**DB**	**OG**	**DC**	**DB**	**OG**	**DC**	**DB**	**OG**	**DC**
Ammonium (μM)	7.5	7.6	4.8	4.1	13.7	4.7	9.7	23.5	22.4
Nitrate (μM)	18.5	19.7	46.2	5.7	15.0	9.4	6.6	4.9	6.7
Nitrite (μM)	0.2	0.3	0.5	0.3	0.6	0.8	0.2	0.6	1.3
Phosphate (μM)	1.3	1.8	1.1	2.4	1.9	1.0	3.0	1.6	1.0
Temperature (°C)	5.2	5	4.9	11.4	8.6	14	15.4	15.4	18.3

### N_2_ fixation in the water column

**Figure 2:**
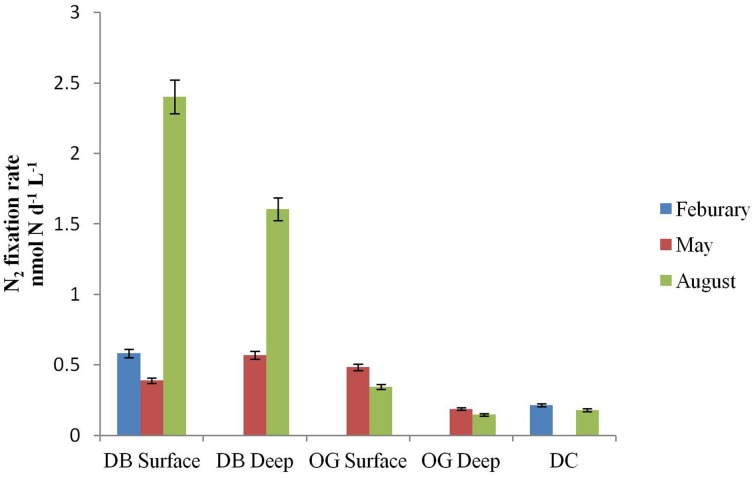
**N_2_ fixation (nmol N L^−1^ d^−1^) in surface and deep water at the stations DB, OG, and DC in February, May and August**.

N_2_ fixation was detected at all stations in the water column as well as in the bottom sediments. In the water column, the rates ranged from 0 to 2.4 nmol N L^−1^ d^−1^ (Figure 2). The highest rate of N_2_ fixation was measured in August at the water surface at station DB. Among the three stations, DB always recorded the highest rate of N_2_ fixation with a peak in August. In February we did not detect N_2_ fixation at station OG and in May not in DC. In August N_2_ fixation was detected in all stations but was highest in station DB.

### Abundance and activity of diazotrophs in the sediment

**Figure 3:**
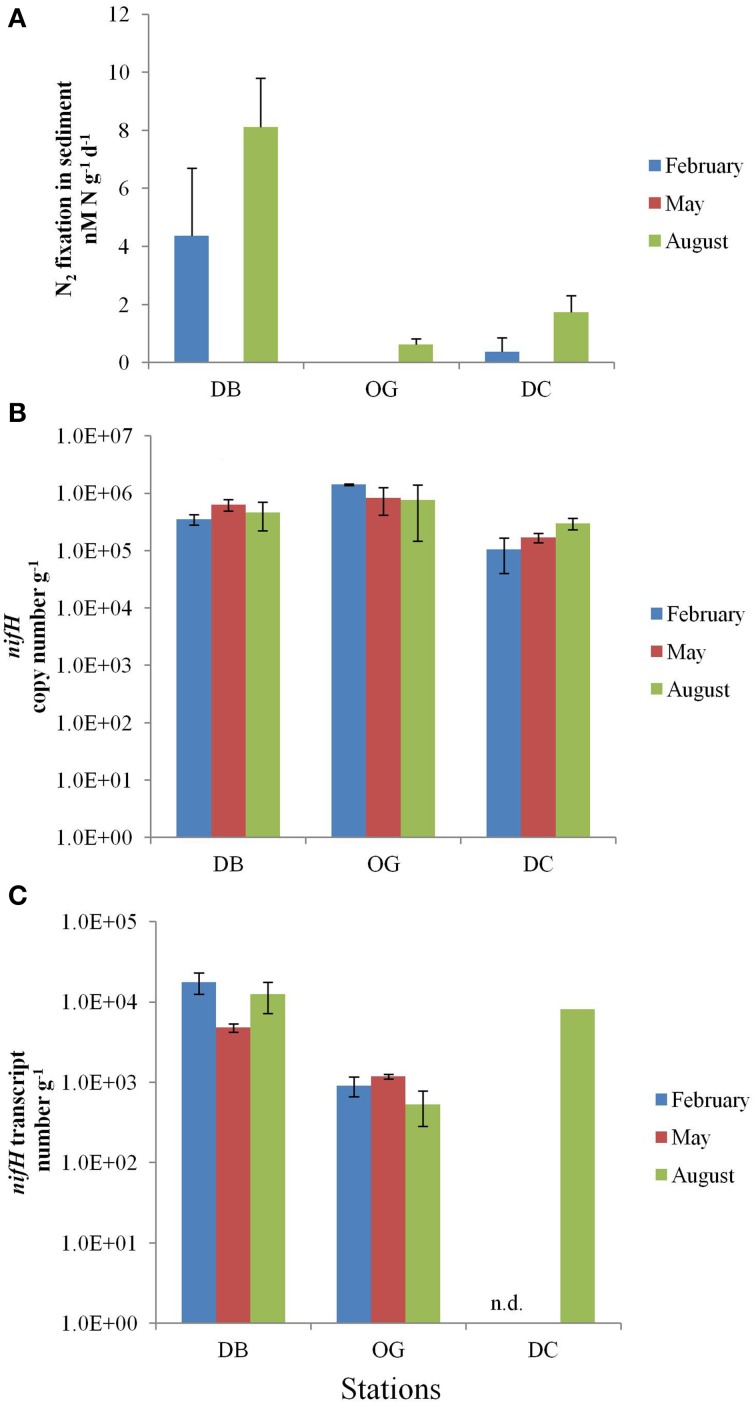
**N_2_ fixation in the bottom sediments at the stations DB, OG, and DC in February, May, and August**. **(A)** potential nitrogen fixation rates (nmol N day^−1^ g^−1^ wet sediment). **(B)** *nifH* copy number (g^−1^ wet sediment). **(C)** *nifH* transcript number (g^−1^ wet sediment). n.d., no data.

In the top 5 cm of the bottom sediments, N_2_ fixation was in the range of 0–8.1 nmol N d^−1^ g^−1^ wet sediment (Figure 3A). The highest rate of N_2_ fixation was recorded in August at station DB. N_2_ fixation was undetectable in February at station OG and at all three stations in May.

The abundance of diazotrophs and denitrifiers was evaluated through the quantification of the *nifH* and *nirS* genes, respectively. Gene copy numbers of *nifH* were more or less constant throughout the seasons. The abundance of *nifH* was highest at station OG (on average 10^6^ copies g^−1^ wet sediment). The stations DB and DC contained on average 4.8 × 10^5^ and 1.9 × 10^5^ *nifH* gene copies g^−1^ wet sediment, respectively (Figure 3B). At all stations expression of the *nifH* gene was detected in the bottom sediment. The values ranged from 5.3 × 10^2^ to 1.8 × 10^4^ and the highest number was recorded in February at station DB (Figure 3C). The number of *nifH* transcripts was 13 times higher at station DB (on average 1.2 × 10^4^ transcripts g^−1^ wet sediment) than that at station OG (on average 8.7 × 10^2^ transcripts g^−1^ wet sediment). We have only data for August at station DC (8.3 × 10^3^ transcripts g^−1^ wet sediment).

### Abundance and activity of denitrifiers in the sediment

**Figure 4:**
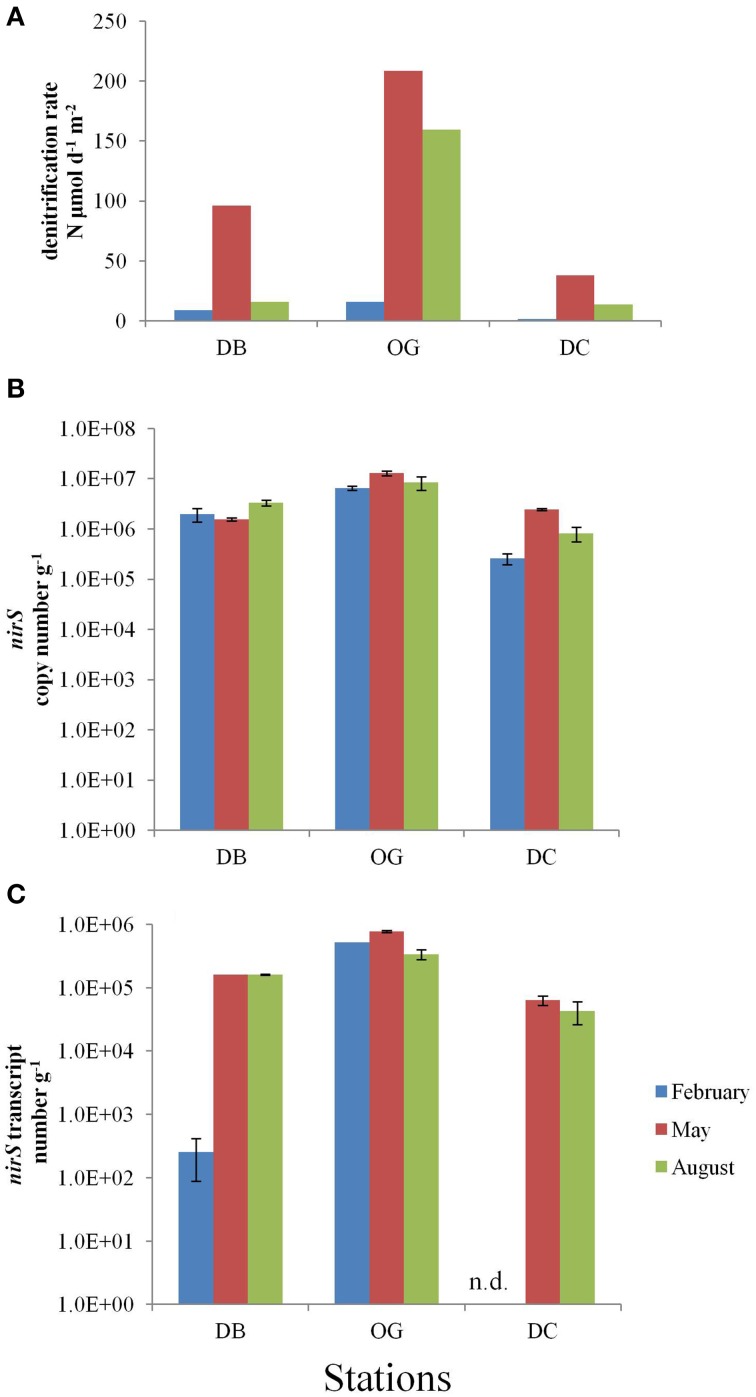
**Denitrification in the bottom sediments at the stations DB, OG, and DC in February, May, and August. (A)** *in situ* denitrification (μmol m^−2^ day^−1^). **(B)** *nirS* copy number (g^−1^ wet sediment). **(C)** *nirS* transcript number (g^−1^ wet sediment). n.d., no data.

Denitrification in the top 10 cm of the bottom sediment was in the range of 1.7–208.8 μmol N m^−2^ d^−1^. The highest rate was recorded in May at station OG and the lowest rate in February at station DC (Figure 4A). Denitrification was always highest at station OG regardless in which of the three seasons studied.

The *nirS* gene abundance followed the same spatial trend as *nifH* and was also highest at station OG, irrespective the season (Figure 4B). At the stations DB and OG the *nirS* gene abundance was more or less constant during the seasons (on average 2.3 × 10^6^ and 9.3 × 10^6^ gene copies g^−1^ wet sediment, respectively), whereas at station DC the numbers increased 9.4-fold between February and May (on average 1.2 × 10^6^ copies g^−1^ wet sediment).

The expression of *nirS* was detected in all samples and ranged 2.5 × 10^2^–7.7 × 10^5^ transcripts g^−1^ wet sediment (Figure 4C). The number of transcripts was highest at station OG (on average 5.4 × 10^5^ transcripts g^−1^ wet sediment) and lowest in DC. We did not observe seasonality at the stations OG and DC whereas the number of transcripts at station DB was three orders of magnitude lower in February than in May and August.

### Diversity, phylogeny, and community composition of diazotrophs and denitrifiers (*nifH* and *nirS* sequences)

**Figure 5:**
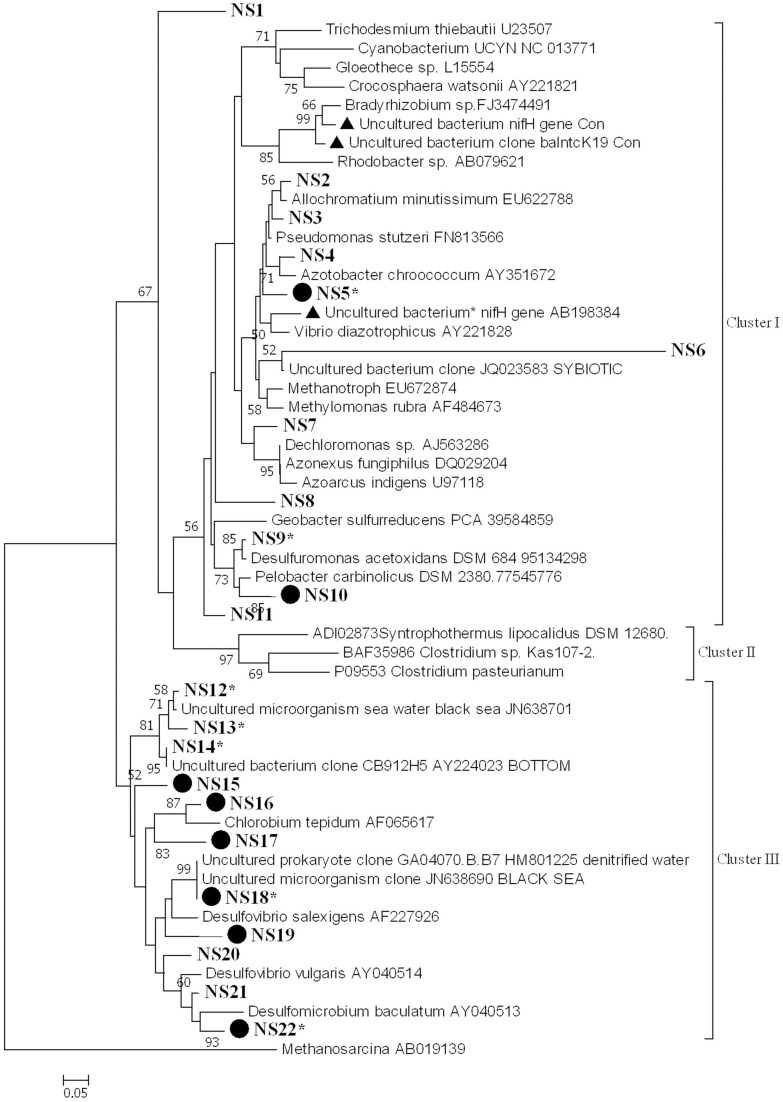
**Phylogenetic tree of NifH based on the translated amino acid sequence, constructed by the neighbor-joining method in MEGA 6**. The scale bar indicates the number of sequence substitution per sites. Sequences retrieved in this study fell into 22 groups (NS1-NS22) and are shown in bold. Table S1 gives all sequences in groups NS1–NS22. Groups that contain sequences from both genomic DNA and cDNA are marked by a solid circle. Asterisks indicate groups that contain sequences from the water column. *nifH* sequences that have been reported previously as potential contaminants in RT-PCR reagents are marked by a solid triangle.

*nifH* diversity and expression were examined in samples collected from the water column (surface and deep) at all stations in August. We examined 15–20 sequences from each library. Amplified *nifH* gene sequences (from DNA and cDNA) fell within the clusters I and III, according to the phylogenetic classification proposed by Zehr et al. (2003) (Figure 5). The cDNA sequences fell mainly into group NS22 (Figure 5). These sequences did not cluster according to stations or to depths. A large proportion of the sequences (60%) belong to cluster III and almost all expressed *nifH* belongs to this cluster.

The diversity of *nifH* and *nirS* was evaluated in sediment samples collected in August. In total 246 sequences were obtained from three *nifH* clone libraries and resulted in 55 OTUs at the 95% identical amino acid level. The coverage of these libraries ranged from 66 to 78%. Based on diversity indices (H, 1/D), the station OG had the lowest diversity and the coastal station DC had highest diversity. The richness estimators S_ACE_ and Chao1 are consistent with these results (Table 2).

**Table 2:** **Biodiversity and predicted richness of the sediment NifH and NirS amino acid sequences from the sampling stations of the Southern North Sea based on 95% cutoffs**.

	**No. of clones**	**No. of OTUs**	**Coverage (%)**	**ACE**	**Chao1**	**Shannon**	**Simpson**
**NifH**							
DB	85	23	73	43	43	2.3	0.21
OG	73	16	78	30	25	1.4	0.50
DC	88	30	66	76	43	2.9	0.09
**NirS**							
DB	33	14	59	47	21	2.3	0.12
OG	40	32	22	719	307	3.2	0.03
DC	45	14	70	25	19	2.0	0.12

Phylogenetic analysis of the deduced amino acid sequences of the *nifH* amplicons revealed that they fell within clusters I and III. Of the 246 *nifH* sequences from the sediment, 63% belonged to cluster I (64, 86, and 42% of the *nifH* sequences from station DB, OG, and DC, respectively). Of these, another 63% could be affiliated to *Pelobacter carbinolicus* (accession number WP_011341851) (48, 86, and 23% of the *nifH* sequences from station DB, OG, and DC, respectively). The rest of the *nifH* sequences belong to cluster III. At all stations *nifH* was expressed and *nifH* sequences from cDNA libraries are subsets of the gene copy libraries.

A total of 118 *nirS* DNA sequences was obtained consisting of 60 OTUs at 95% identity at the amino acid level. The coverage ranged from 22 to 70% with the poorest one observed at station OG. In contrast to *nifH*, the highest diversity and richness were found at station OG while the lowest values were found at station DC (Table 2).

**Figure 6:**
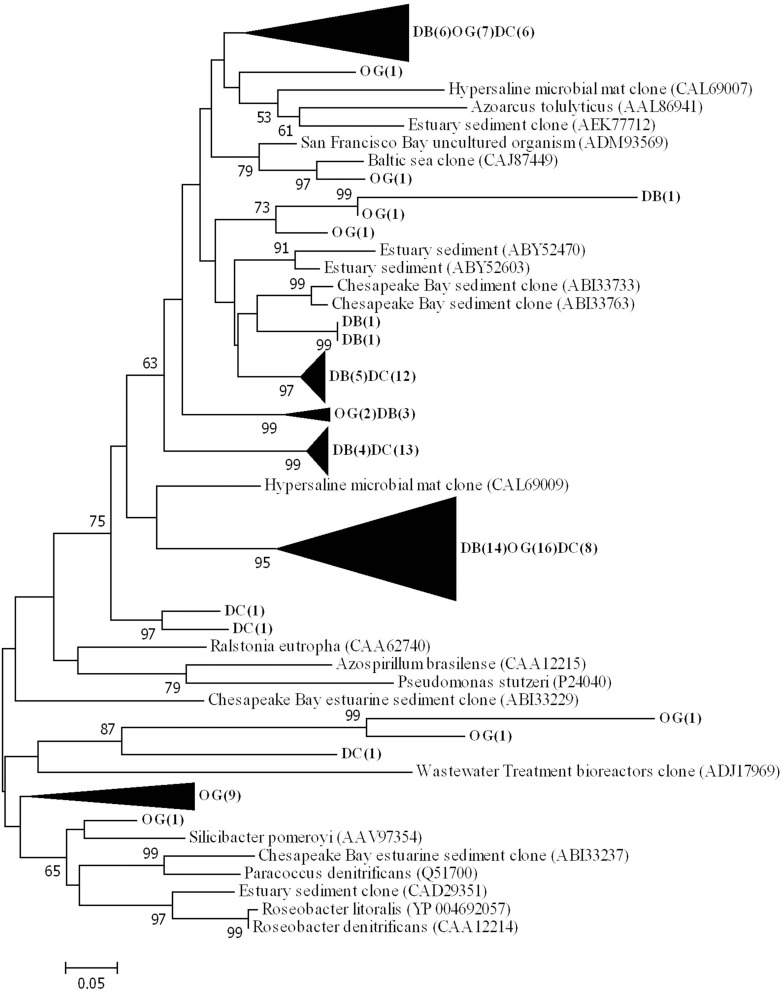
**Phylogenetic tree of NirS based on the translated amino acid sequence, constructed by the neighbor-joining method in MEGA 6**. The scale bar indicates the number of sequence substitution per sites. Sequences retrieved in this study are shown in bold. The numbers in the parenthesis following the station name indicate the number of sequences within this cluster at that station.

Phylogenetic analysis of the deduced amino acid sequences for *nirS* gene fragments showed that the majority of sequences clustered and are related to environmental clones from a variety of environments (e.g., ABI33733 from Chesapeake Bay, CAL69007 from a hypersaline microbial mat and CAJ87449 from the Baltic Sea) (Figure 6). The sequences were closest related to those belonging to *Thiothrix lacustris* (AGO45492) (similarity 83%) *Azoarcus tolulyticus* (AAL86941) (similarity 72%).

**Figure 7:**
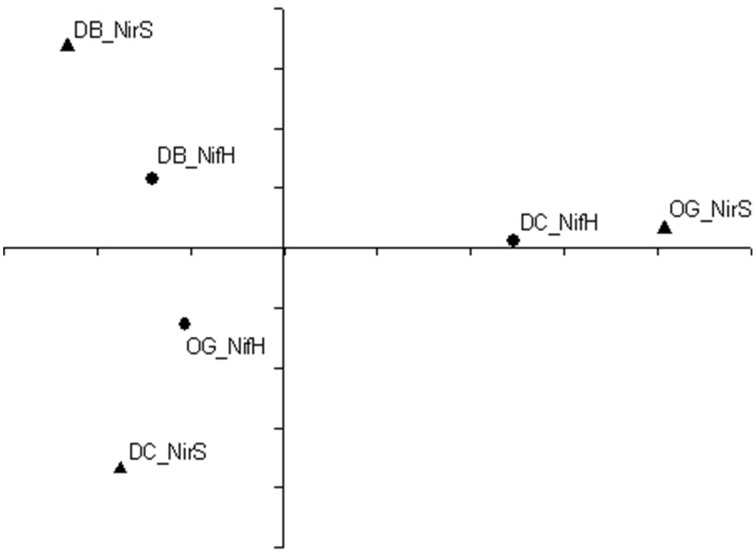
**Principal coordinate analyses of** ***nifH*** **(solid circle) and** ***nirS*** **(solid triangle) translated amino acid sequences at the 95% cutoff**.

Principal coordinate analysis was performed to show the differences in composition of diazotrophic and denitrifying communities between stations. As shown in Figure 7, the diazotrophic community composition at station DC was different from the other two stations. The denitrifying community compositions at station DB and station DC were more similar to each other than to station OG.

## Discussion

Recent studies expanded biological N_2_ fixation to include temperate coastal waters (Rees et al., 2009; Mulholland et al., 2012). The nitrogen fixation rates (0–2.4 nmol N L^−1^ d^−1^) in the water column of the southern North Sea that we report here were in the same range as those that have been reported for the tropical Atlantic Ocean (0.6–1.1 nmol N L^−1^ d^−1^) (Falcón et al., 2004), the subtropical and tropical eastern Atlantic Ocean (0–1.4 nmol N L^−1^ d^−1^) (Staal et al., 2007) and from some stations of the western Atlantic coastal waters (0.2–76.8 nmol N L^−1^ d^−1^) (Mulholland et al., 2012) but substantially lower than those reported for the western English Channel (18.9–20.0 nmol N L^−1^ d^−1^) (Rees et al., 2009). Depth integrated rates of nitrogen fixation ranged from 1.25 to 62.5 μmol N m^−2^ d^−1^ at the stations of the southern North Sea. These rates of N_2_ fixation were detected throughout the year but may appear low relative to other coastal ecosystems such as microbial mats, coral reefs, sea grass meadows, and intertidal marshes. N_2_ fixation also occurred in the bottom sediments of the stations. This is the first time that N_2_ fixation is reported from the water column and bottom sediments in the cold waters of the North Sea. The rates of denitrification that we observed in the North Sea (1.7–208.8 μmol N m^−2^ d^−1^) compared well with other reports [240–320 μmol N m^−2^ d^−1^, (Lohse et al., 1996) and 700 μmol N m^−2^ d^−1^, Hydes et al., 1999]. When comparing the rates of N_2_ fixation and denitrification we conclude that these processes seem to be in balance.

Sequence analysis revealed that *nifH* expressed in the water column belong to clusters I and III. Cluster I sequences that are closely related to delta-proteobacteria have been found in the English Channel (EF470531) (Rees et al., 2009). Cluster III sequences affiliated to *Desulfovibrio salexigens* have been found in the western mid-Atlantic coastal waters (FJ756655) (Mulholland et al., 2012). Hence, these diazotrophs seem to be common in Atlantic coastal waters. We did not found *nifH* sequences belonging to cyanobacteria or γ-proteobacteria although these groups are regarded as the dominant diazotrophs in the marine environment (Capone et al., 1997; Montoya et al., 2004; Halm et al., 2012; Moisander et al., 2014) and were also detected in English Channel (Rees et al., 2009). The absence of these groups would explain the low rates of N_2_ fixation rates measured in our study compared to those recorded in the western English Channel. The fact that the *nifH* sequences retrieved in oxygenated surface water hints to the presence of anaerobic groups requires an explanation. Given that some of the retrieved *nifH* phylotypes have also been reported from coastal microbial mats (Severin et al., 2010), we speculate that benthic microorganisms might have been brought into suspension. But we cannot exclude the possibility that anaerobic diazotrophs thrive in anoxic microniches such as in aggregates (Ploug, 2001), or that the sequences belong to other aerobic organisms.

A large proportion of *nifH* homologs obtained from the sediment belongs to *P. carbinolicus*, similar to what has been found in other studies (Fulweiler et al., 2013; Brown and Jenkins, 2014). *P. carbinolicus* couples the oxidation of organic matter or metals to the dissimilatory reduction of Fe(III) or S° (elemental sulfur) (Lovley et al., 1995; Holmes et al., 2004). *P. carbinolicus* is a strictly anaerobic bacterium belonging to the deltaproteobacterial family of *Peleobacteraceae*. Organisms of this family are known for their bioremediation potential. It is currently unknown whether this organism fixes N_2_ but genome sequencing revealed the presence of genes encoding proteins that are involved in nitrogen fixation including a cluster containing *nifHDK* homologs, genes involved in molybdenum biosynthesis and several other nif-genes (Aklujkar et al., 2012). There are also no reports of studies on N_2_ fixation by *P. carbinolicus* although the phylogenetically related *Geobacter metallireducens* has been shown to fix N_2_ (Bazylinski et al., 2000). Holmes et al. (2004) found that N_2_ fixation is a highly conserved trait in the *Geobacteraceae* and proposed that it gives the members of this family the advantage to compete in environments that are being bio-remediated. But it remains to be seen whether this is also the case in *P. carbinolicus*.

The sequencing results suggest that sulfate reducers such as *D. salexigens* and *Desulfovibrio vulgaris* may be the dominant diazotrophs in the bottom sediments of the southern North Sea. Many sulfate-reducing bacteria possess the genetic potential to fix dinitrogen (Zehr et al., 1995). These diazotrophic organisms were also found in the sediments of the Baltic Sea (Bertics et al., 2013), Narragansett Bay (Fulweiler et al., 2013), and coastal California (Bertics et al., 2010). Previous studies found that nitrogenase activity decreased substantially when sulfate reduction was inhibited (Burns et al., 2002; Bertics et al., 2013). In addition, Bertics et al. (2013) and Brown and Jenkins (2014) complemented these findings with genetic data showing that the *nifH* sequences retrieved were closely related to sulfur and sulfate reducers *Desulfovibrio* and *Desulfobacter* spp. This supports the idea that sulfate-reducing bacteria are key players in sedimentary N_2_ fixation.

It has been shown that denitrification and nitrogen fixation are both controlled by the same common factors such as temperature, oxygen, and substrate availability (e.g., organic matter and nitrite for denitrification) (Joye and Paerl, 1994; Nowicki et al., 1997; Kana et al., 1998; Staal et al., 2003; Fulweiler et al., 2007). In this study we observed different seasonality and spatiality of sedimentary nitrogen fixation and denitrification, suggesting that the individual response is different.

Spatial variation of denitrification is primarily attributed to the distribution of organic carbon in the bottom surface sediments. Trimmer and Nicholls (2009) have shown that sedimentary denitrification correlated positively with the concentration of organic carbon in the surface sediments along a transect in the North Atlantic. Station OG is a recognized deposition area with muddy sands and contains an order of magnitude more organic carbon compared to the other two stations (Bale et al., 2013). This would explain the higher rate of denitrification at station OG. Temporally, denitrification was highest in spring and this seasonality coincided well with the abundance of the *nirS* gene transcripts. This result is also consistent with a previous study in the southern North Sea. Van Raaphorst et al. (1992) estimated the denitrification at two stations in the southern North Sea and showed that denitrification was highest in spring and early summer and lowest in winter. Different seasonality of denitrification has also been observed in other studies. For instance, Tuominen et al. (1998) reported highest denitrification in Baltic Sea bottom sediments in late summer and early autumn while Hietanen and Kuparinen (2008) observed the highest rates in autumn and early winter in the sediment in the Gulf of Finland. High rates of denitrification in late spring could be attributed to an elevated temperature and an increased supply of fresh organic carbon deposited from spring blooms (Joint and Pomroy, 1993). Moreover, during late spring and summer, the increased availability of organic matter would stimulate the consumption of O_2_, which would enhance denitrification. The bottom water at station OG can become hypoxic in summer (Weston et al., 2008; Greenwood et al., 2010), and consequently this would decrease the oxygen penetration depth in the sediment. The low oxygen concentration in the sediment at station OG may be responsible for the observed high rate of denitrification. In addition, a previous study has shown that nitrification is also higher in summer than in winter in the North Sea sediment (Lohse et al., 1993). When the source of nitrate/nitrite in the water column is limited in summer (nitrate/nitrite concentrations in bottom water have been reported in Bale et al., 2013), nitrification becomes the primary source of nitrate/nitrite for denitrification.

In contrast to denitrification, station DB exhibited the highest pelagic and sedimentary rates of nitrogen fixation as well as the highest number of *nifH* transcripts when compared to the other two stations. Station DB is located at the shallow Dogger Bank. Previous studies indicated that the Dogger Bank is a special ecological area with distinct biological characteristics compared to the surrounding regions in the North Sea (Kröncke and Knust, 1995). Throughout the year the Dogger Bank exhibits high rates of primary production (Howarth et al., 1993). This introduces fresh organic matter to the water column and the bottom sediment, fuelling heterotrophic N_2_ fixation. This would explain why N_2_ fixation occurred even in February in the water column and bottom sediment at the sandy station DB. O'Neil and Capone (1989) found that N_2_ fixation in coarse-grained marine sediments is higher in eutrophic than in oligotrophic environments and is generally stimulated by the addition of organic matter.

In this study we observed that N_2_ fixation is generally higher in summer than in winter and this also the case for denitrification for the same reasons as discussed above. The exception was that benthic N_2_ fixation was undetectable in spring (May) when denitrification was highest. This difference suggests that N_2_ fixation and denitrification respond differently to the post bloom deposition. Fulweiler et al. (2007) showed that denitrification responded rapidly and positively to the deposition of organic matter. Van Luijn et al. (1999) investigated nitrogen fluxes and processes in the bottom sediments of a shallow eutrophic lake and found that denitrification increased with increasing contents of fresh organic matter but then deceased when a certain concentration of organic matter was exceeded. These observations coincided well with our seasonal denitrification trend. It is not clear why N_2_ fixation did not respond to the post bloom deposition in spring. Fulweiler et al. (2013) proposed that the quality of organic matter plays a role in controlling N_2_ fixation. It is possible that a different timing of the phytoplankton blooms alters both the quantity and quality of the deposited organic matter (Nixon et al., 2009) and we speculate that benthic diazotrophs depend on a restricted range of organic matter and/or its concentration. The positive correlation (Pearson, ρ = 0.95, *p* < 0.05, *n* = 9) between pelagic and benthic N_2_ fixation suggests that both respond in the same way to environmental factors.

The spatial and temporal separation of denitrification and nitrogen fixation is also projected on the composition of chemotrophic diazotrophic and denitrifying communities. We hypothesize that the characteristics of the sediment may be a factor that determines the diazotrophic and denitrifying community compositions. Both the abundances of *nifH* (Pearson, ρ = 0.82 *p* < 0.05, *n* = 9) and *nirS* (Pearson, ρ = 0.90, *p* < 0.05, *n* = 9) genes are positively correlated with total organic matter (Bale et al., 2013). However, the diazotrophic community composition at the three stations appears to correlate with geographic location (coastal and offshore) rather than with sediment type (sandy and muddy). In contrast, sediment type determined denitrifying community composition (based on *nirS* gene). The community composition of denitrifiers at station DB and station DC are more similar to each other than to station OG. Low organic carbon content and a large grain size are characteristic for the former two stations (Bale et al., 2014).

High abundance of *nifH* copies at station OG indicated the genetic potential of N_2_ fixation at this station. Nevertheless, the number of *nifH* transcripts was low and we did not find N_2_ fixation at station OG while the opposite was true for station DB. There are several explanations for this discrepancy. First, heterotrophic N_2_ fixers may have to compete for carbon with other heterotrophic microorganisms such as denitrifiers, which were highly active at station OG. Second, the quality and quantity of organic matter may determine the activity of diazotrophs. It has been shown that the water mass at the three stations are not the same (Bale et al., 2013). This could lead to a deposition of organic matter that differs in quality and quantity. Third, although organic matter would promote oxygen consumption in sediment, the degradation of organic matter also leads to the accumulation of nitrate and ammonium, which could lead to inhibition of N_2_ fixation. Whether or not this latter possibility is realistic is unclear. In this study as well as in other published reports, N_2_ fixation took place in the presence of considerable levels of dissolved inorganic nitrogen (DIN). The thresholds of DIN at which N_2_ fixation under natural conditions is inhibited have not been precisely determined. N_2_ fixation activity has been detected in diverse pelagic and benthic environments while the DIN concentrations were higher than those in our study. Mulholland et al. (2012) found N_2_ fixation in the presence of DIN and low phosphate concentrations in a temperate marine system. Haines et al. (1981) found N_2_ fixation in a coastal sediment in Alaska in the presence of an ammonium concentration of 177 μM. Bertics et al. (2010) detected nitrogenase activity in the subsurface of bioturbated sediments when ambient ammonium concentrations were >50 μM and Bertics et al. (2013) even detected N_2_ fixation by sulfate reducing bacteria in the presence of 1 mM ammonium. However, contrary to these observations, Joye and Paerl (1994) found that N_2_ fixation decreased when ambient DIN concentrations increased. Similar observations of N_2_ fixation in sea-grass-bearing sediments coincided with an annual minimum ammonium concentration of 190 μM (Welsh et al., 1996). We found no evidence of inhibition of N_2_ fixation by DIN and this is consistent with other studies (Haines et al., 1981; Bertics et al., 2010, 2013). Nitrogen fixation may also benefit from high phosphate concentration. It has been shown that DIN may not be inhibiting N_2_ fixation in the euphotic zone of marine waters, especially when phosphate and trace metals are abundant (Knapp, 2012, and references therein). The positive correlation (Pearson, ρ = 0.84, *p* < 0.05, *n* = 9) between benthic N_2_ fixation and phosphate concentration in our study supports the view that phosphate plays a crucial role. It is not clear why the energetically expensive N_2_ fixation occurs while sufficient DIN is available. This would be understandable when N_2_ fixation would serve another function such as an electron sink as suggested by Tichi and Tabita (2000). Moreover, even at high bulk ammonium concentrations N_2_ fixation may be favored in microzones that are depleted of ammonia for instance because ammonia oxidizers decrease the concentration of ammonium locally and there may be also other microorganisms that compete for this source of nitrogen.

In conclusion, N_2_ fixation and denitrification were temporally and spatially separated. The former was highest in August in the DB station, a sandy area with low organic content, while the latter was high in May in the OG station, a muddy depression in the North Sea with high organic content. Nevertheless, both processes were more or less in balance. The rates of both processes coincided with the expression of the functional genes *nifH* and *nirS*, but not with the number of gene copies present. A high number of gene copies indicated the potential for N_2_ fixation and denitrification but was not a good indicator of the actual process. N_2_ fixation was mainly attributed to the anaerobic sulfate reducing bacteria. The functional gene representing denitrification, *nirS*, could not be assigned to a specific group of microorganisms.

### Conflict of interest statement

The authors declare that the research was conducted in the absence of any commercial or financial relationships that could be construed as a potential conflict of interest.
