# Supplementary material for: Drivers of the dynamics of diazotrophs and denitrifiers in North Sea bottom waters and sediments
Source: Front Microbiol. 2015 Jul 21;6:738. doi: 10.3389/fmicb.2015.00738 (PMC4508842; doi:10.3389/fmicb.2015.00738)
Supplement: Supplementary file 1 [file Table1.DOCX]

Table S1. List of *nifH* gene sequences from this study.

| Group Name | Sequence sources | Gen Bank accession numbers |  |
| --- | --- | --- | --- |
| NS1 | DC | KP959586 |  |
| NS2 | DC | KP959524, KP959534 |  |
| NS3 | DB | KP959426 |  |
| NS4 | DC | KP959560 |  |
| NS5 | DB,DC,WATER | KP959367*,KP959375,KP959387,KP959390,KP959549*,KP959597, KP959731 |  |
| NS6 | DC | KP959593 |  |
| NS7 | DC | KP959518, KP959560, KP959567, KP959569 |  |
| NS8 | DB | KP959422 |  |
| NS9 | WATER | KP959729 |  |
| NS10 | DB, OG,DC, WATER | KP959349-KP959356*, KP959359-KP959363, KP959369, KP959374-KP959375,KP959378, KP959381-KP959382, KP959384-KP959386, KP959389,KP959392-KP959394, KP959396-KP959397, KP959399, KP959402,KP959404-KP959406, KP959410-KP959411, KP959413-KP959416, KP959430-KP959431,KP959434-KP959436, KP959438-KP959440, KP959442-KP959445, KP959447, KP959449  -KP959467, KP959470-KP959473, KP959475-KP959478,KP959480, KP959483-KP959494, KP959497-KP959504, KP959506-KP959508, KP959510-KP959511, KP959513，KP959515, KP959522, KP959527, KP959536-KP95953, KP959539, KP959542, KP959545,  KP959552-KP959553, KP959556, KP959564-KP959566, KP959568, KP959571, KP959577,KP959582, KP959584, KP959588, KP959590, KP959721-KP959722, KP959728-KP959729, KP959733,  KP959349-KP959356, KP959359-KP959363, KP959369, KP959374-KP959375, KP959378, KP959381-KP959382, KP959384-KP959386, KP959389, KP959392-KP959394, KP959396-KP959397, KP959399, KP959402,KP959404-KP959406, KP959410-KP959411, KP959413-KP959416*, KP959430-KP959431,KP959434-KP959436,KP959438-KP959440, KP959442-KP959445, KP959447, KP959449-KP959467, KP959470-KP959473, KP959475-KP959478, KP959480*, KP959483-KP959494*, KP959497-KP959504, KP959506-KP959508, KP959510-KP959511, KP959513, KP959515, KP959522, KP959527, KP959536-KP959537, KP959539*, KP959542, KP959545, KP959552-KP959553, KP959556,KP959564-KP959566*, KP959568, KP959571, KP959577,KP959582, KP959584, KP959588, KP959590, KP959721-KP959722,KP959728-KP959729,KP959733* |  |
| NS11 | DB | KP959378, KP959381-KP959382, KP959384-KP959386, KP959389 |  |
| NS12 | WATER | KP959723 |  |
| NS13 | WATER | KP959730 |  |
| NS14 | WATER | KP959724 |  |
| NS15 | OG,DC | KP959474*, KP959482, KP959495-KP959496, KP959509*, KP959516, KP959519,KP959523, KP959525, KP959528-KP959529, KP959533, KP959535, KP959543, KP959546-KP959548,KP959554-KP959555, KP959557-KP959559, KP959572*, KP959574- KP959576*, KP959578-KP959579, KP959581, KP959583*, KP959587*, KP959589*, KP959591-KP959592, KP959594-KP959595 |  |
| NS16 | DB | KP959365*, KP959368, KP959391, KP959398, KP959409, KP959419 |  |
| NS17 | DB, OG, DC | KP959366*, KP959377, KP959379, KP959417-KP959418*, KP959420-KP959421, KP959428, KP959433, KP959468, KP959526, KP959541, KP959596* |  |
| NS18 | WATER | KP959732 |  |
| NS19 | DB, DC | KP959358*, KP959371-KP959372, KP959380*, KP959383, KP959387*, KP959395*, KP959407, KP959427, KP959520-KP959521 |  |
| NS20 | DC | KP959531 |  |
| NS21 | DB,DC | KP959423, KP959540, KP959563, KP959580 |  |
| NS22 | DB,OG, DC, WATER | KP959364*, KP959388, KP959400, KP959469, KP959505, KP959514, KP959530, KP959544, KP959550, KP959570, KP959573, KP959585*, KP959599- KP959600*, KP959719- KP959720, KP959725- KP959727* |  |
|  |  |  |  |

* sequences detected in both DNA and cDNA. WATER indicates sequences from the water column
